# Erdosteine modulates surfactant-associated markers and preserves lung architecture in a preterm rat model compared with dexamethasone and betamethasone

**DOI:** 10.1038/s41598-026-54256-y

**Published:** 2026-05-28

**Authors:** Serife Ozlem Genc, Caglar Yildiz, Mahmut Sahin, Alper Serhat Kumru, Mustafa Özkaraca

**Affiliations:** 1 Department of Obstetrics and Gynecology, Sivas Cumhuriyet University Faculty of Medicine, Sivas, Turkey; 2 Faculty of Veterinary Medicine, Department of Veterinary Pharmacology and Toxicology, Sivas, Turkey; 3 Faculty of Veterinary Medicine, Department of Veterinary Pathology, Sivas Cumhuriyet University, Sivas, Turkey

**Keywords:** Preterm birth, Antenatal corticosteroids, Erdosteine, Lung maturation, Surfactant proteins, Inflammation, Diseases, Medical research, Physiology

## Abstract

Preterm birth remains a major contributor to neonatal morbidity, primarily due to immature lung development and insufficient surfactant production. Although antenatal corticosteroids (ACS) are widely used to accelerate fetal lung maturation, concerns regarding their potential systemic effects have prompted the search for alternative or adjunctive agents. Erdosteine, a thiol-based compound with anti-inflammatory and antioxidant properties, may represent a promising candidate. Pregnant Sprague–Dawley rats were randomly assigned to four groups (*n* = 6 dams per group): control, dexamethasone (DEX), betamethasone (BET), and erdosteine (ERDO). All treatments were administered antenatally to the pregnant dams via intraperitoneal injection. DEX and BET were given on gestational days (GD) 16–18, while ERDO was administered on GD16–20. Preterm delivery was achieved by cesarean section on GD20. Lung tissues of pups born to treated dams were collected on postnatal day 5 (P5) and analyzed using histopathological and immunohistochemical methods, including Caspase-3, PCNA, ABCA3 (ATP-binding cassette subfamily A member 3), SFTPA1, AQP5, and SP-B. DEX and BET groups demonstrated lung architecture consistent with accelerated maturation, whereas ERDO preserved alveolar structure with only mild interstitial inflammation. Caspase-3 and PCNA expression were markedly increased in corticosteroid-treated groups but remained low in ERDO and control groups. Notably, ABCA3 expression was highest in the ERDO group across both compartments (*p* < 0.001). SFTPA1 expression was also more prominent in the interstitial compartment in the ERDO group. AQP5 and SP-B showed no significant expression in any group. Erdosteine demonstrated a biologically favorable profile characterized by reduced inflammatory response, preservation of lung architecture, and enhanced expression of key surfactant-related markers, particularly ABCA3. While not fully replicating the effects of antenatal corticosteroids, its anti-inflammatory and antioxidant properties suggest that it may serve as a supportive or complementary strategy for promoting fetal lung maturation. Further experimental and translational studies are warranted.

## Background

Preterm birth accounts for nearly 11% of all live births and remains a leading contributor to neonatal morbidity and mortality worldwide^1,2^. Respiratory distress syndrome, primarily driven by inadequate lung maturation and surfactant deficiency, represents one of the most critical complications associated with early delivery^3^. For decades, antenatal corticosteroids (ACS) have been the cornerstone of perinatal management, significantly reducing respiratory morbidity by accelerating structural and functional maturation of the fetal lung^4^. Their primary mechanism involves promoting type II pneumocyte differentiation and enhancing the expression of key surfactant-associated proteins (SP-A, SP-B, SP-C), as well as ABCA3, a crucial regulator of phospholipid transport and lamellar body formation^5^.

Despite these well-established benefits, increasing concern has emerged regarding the potential long-term systemic effects of antenatal corticosteroid exposure. Accumulating evidence suggests that prenatal glucocorticoids may induce persistent developmental programming in extra-pulmonary organs, including alterations in the hypothalamic–pituitary–adrenal axis^6–8^, as well as possible adverse effects on neurodevelopmental and cardiometabolic outcomes^9^. Experimental studies further indicate that prenatal dexamethasone exposure may disrupt gonadal development, impair testicular barrier integrity, and alter ovarian structure, highlighting the broader spectrum of glucocorticoid-related developmental toxicity^10–12^. These concerns underscore the need for alternative or adjunctive strategies that can support lung maturation while minimizing systemic endocrine and developmental risks.

Within this context, erdosteine has emerged as a biologically plausible candidate. Erdosteine is a thiol-containing prodrug that is metabolized into active free-sulfhydryl derivatives, particularly the M1 metabolite, which exert potent antioxidant and anti-inflammatory effects^13^. Through modulation of oxidative stress and inflammatory pathways, erdosteine has demonstrated protective effects in various models of lung injury, suggesting a potential role in preserving alveolar integrity and supporting pulmonary development^14,15^. However, despite its favorable safety profile and established pulmonary effects, its potential contribution to fetal or early neonatal lung maturation has not yet been investigated.

Therefore, the present study aimed to evaluate the effects of antenatal erdosteine administration on preterm lung maturation in a rat model and to compare its impact with that of dexamethasone and betamethasone, with a particular focus on surfactant-associated markers and compartment-specific histopathological changes.

## Methods

### Study design and ethical approval

This controlled experimental study was designed to compare the effects of erdosteine with dexamethasone (DEX) and betamethasone (BET) on preterm lung maturation.

All experimental procedures were approved by the Sivas Cumhuriyet University Animal Experiments Local Ethics Committee (HADYEK) (Decision No: 658, dated 24.11.2023) and conducted in accordance with international guidelines for animal research, including the Guide for the Care and Use of Laboratory Animals.

The study was also performed in compliance with the ARRIVE 2.0 guidelines.

An a priori power analysis was not feasible due to the exploratory nature of the study; however, group sizes were consistent with previously published antenatal corticosteroid–surfactant rat models, ensuring biological validity.

### Animals and confirmation of pregnancy

Female Sprague–Dawley rats weighing 230–250 g were used. Animals were acclimatized under standard laboratory conditions.

Animals were housed under controlled environmental conditions (22 ± 2 °C, 50–60% relative humidity, 12-hour light–dark cycle) with ad libitum access to food and water.

All husbandry procedures conformed to the *Guide for the Care and Use of Laboratory Animals* and adhered to AAALAC (Association for Assessment and Accreditation of Laboratory Animal Care) International standards.

Estrous cycle synchronization was performed using daily vaginal smear analysis. Females exhibiting cornified epithelial cells were considered to be in the estrus phase and selected for mating.

Female rats were housed with males overnight for mating. Pregnancy was confirmed by the presence of a vaginal copulatory plug, and this day was designated as gestational day 0 (GD0). Pregnant rats were then separated and allocated to experimental groups.

On gestational day 20, cesarean section was performed under appropriate anesthesia to obtain preterm pups. To ensure postnatal survival and appropriate care following cesarean delivery and maternal euthanasia, a cross-fostering approach was implemented. Additional pregnant dams were synchronized to deliver naturally on the same day. Immediately after cesarean delivery, pups from the experimental groups were transferred to lactating surrogate dams that had delivered within a few hours^16^.

Prior to transfer, pups were exposed to the bedding material of the foster dam to facilitate olfactory adaptation and maternal acceptance. The biological pups of the surrogate dams were removed to standardize litter size and ensure adequate access to maternal milk.

All fostered pups were naturally breastfed by the surrogate dams and maintained under standard laboratory conditions throughout the postnatal period. Pup survival, feeding behavior, and general activity were monitored daily until postnatal day 5 (P5), at which point tissue collection was performed.

Following delivery, dams were euthanized under deep anesthesia using an intraperitoneal injection of sodium pentobarbital (200 mg/kg). Adequate depth of anesthesia was confirmed by the absence of corneal and pedal withdrawal reflexes prior to euthanasia. This method was selected in accordance with institutional animal care guidelines to ensure rapid and humane death.

### Experimental groups and randomization

Pregnant rats were randomly assigned into four groups (*n* = 6 dams per group): Control, DEX, BET, and ERDO.

Each dam was considered an independent experimental unit.

For histopathological and immunohistochemical analyses, pups were selected from different litters to avoid litter-related bias, and no more than one pup per litter was included in each analysis.

### Drug administration

All pharmacological agents were administered to the pregnant dams via intraperitoneal injection.

- Control group: Received 2 mL saline on gestational days (GD) 16, 17, and 18.
- DEX group: Received dexamethasone at a dose of 1.0 mg/kg on GD16, followed by 0.5 mg/kg on GD17 and GD18.
- BET group: Received betamethasone at a dose of 0.25 mg/kg on GD16, followed by 0.125 mg/kg on GD17 and GD18.
- ERDO group: Received erdosteine at a dose of 150 mg/kg/day on GD16–20.

Drug doses were selected based on previously published studies^12,17,18^.

### Tissue collection and histopathological evaluation

On postnatal day 5 (P5), pups born to treated dams were euthanized with a high-dose intraperitoneal injection of pentobarbital. Lung tissues were inflation-fixed with 10% neutral-buffered formalin, processed routinely, embedded in paraffin, and sectioned at a thickness of 5 μm for histopathological and immunohistochemical analyses.

Hematoxylin and eosin (H&E) staining was performed on lung sections. A blinded pathologist evaluated the following parameters:

- Alveolar structural maturation.
- Interstitial thickness.
- Degree of mononuclear inflammatory infiltration.
- Overall architectural integrity.

**Fig. 1:**
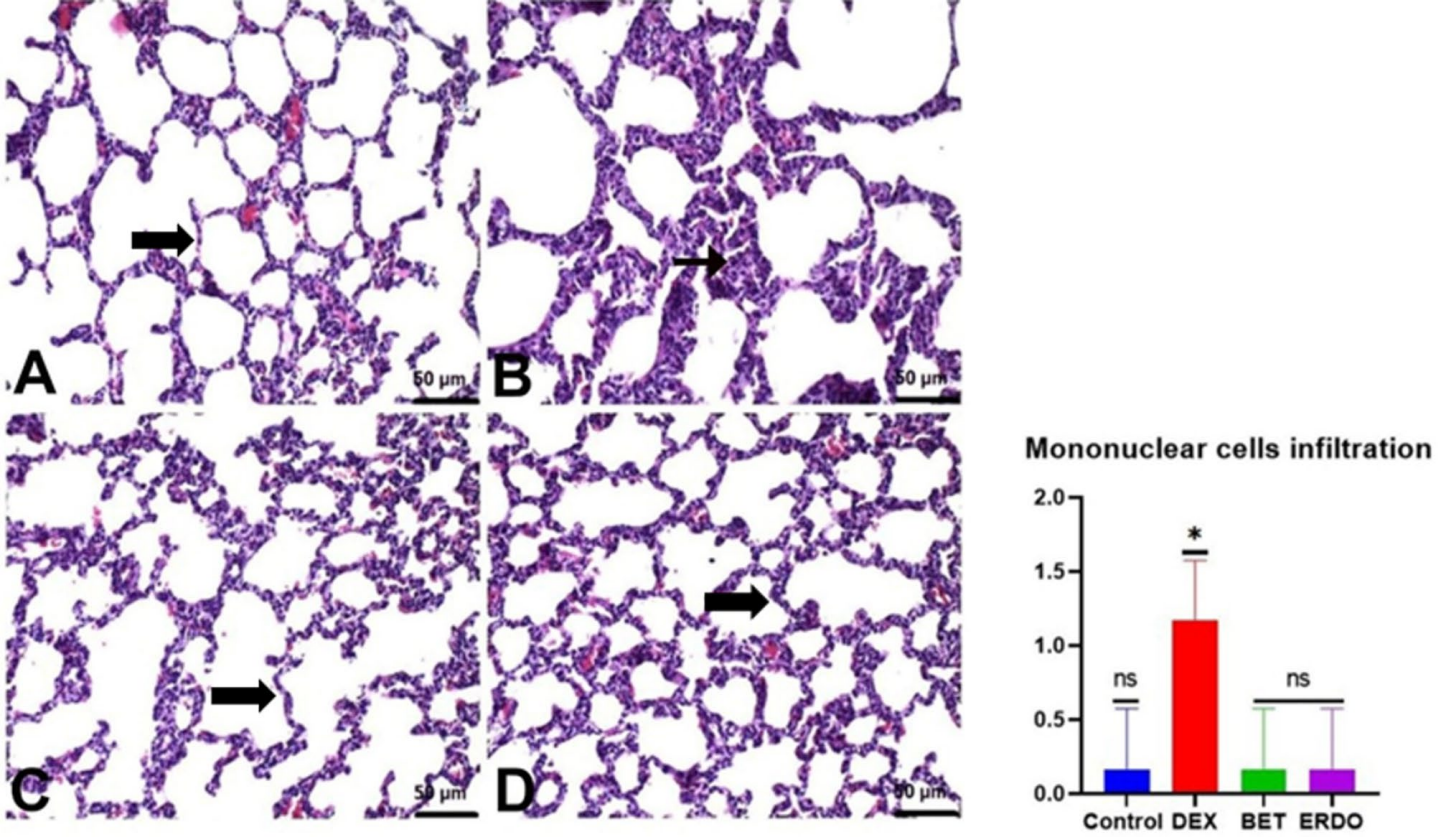
Representative H&E-stained sections of lung tissue from Control (**A**), ERDO (**B**), BET (**C**), and DEX (**D**) groups, along with semi-quantitative analysis of mononuclear cell infiltration. Arrows indicate areas of mononuclear inflammatory cell infiltration in the ERDO group and representative regions with minimal or absent inflammatory cells in the Control, DEX, and BET groups to facilitate visual comparison. Bar graph shows semi-quantitative scoring of mononuclear cell infiltration (0 = none, 1 = mild, 2 = moderate).Statistical significance is indicated (*p < 0.05; ns: not significant). Scale bar: 50 μm.

A semi-quantitative scoring system was applied for mononuclear cell infiltration as follows: 0 = none, 1 = mild, 2 = moderate. Representative micrographs are presented in Fig. 1.

### Immunohistochemistry

Immunohistochemical staining was performed on lung tissue sections obtained from pups born to treated dams. The following primary antibodies were used:

- Caspase-3 (apoptosis).
- PCNA (proliferation).
- ABCA3 (surfactant phospholipid transporter).
- SFTPA1 (surfactant protein A1).
- AQP5 (type I pneumocyte marker).
- SP-B (surfactant protein B).

Details of the antibodies, including manufacturer and catalog numbers, are provided in Table 1.

**Table 1:** Antibodies Used in Immunohistochemistry.

Marker	Manufacturer	Catalog no.
Caspase-3	ThermoFisher	PA5-114687
PCNA	Abcam	ab29
ABCA3	Affbiotech	DF9245
SFTPA1	Affbiotech	DF7204
AQP5	Affbiotech	AF5169
SP-B	Affbiotech	DF8615

Following deparaffinization and antigen retrieval using citrate buffer (pH 6.0), sections were incubated with primary antibodies overnight at 4 °C. Detection was performed using horseradish peroxidase (HRP)-conjugated secondary antibodies and 3,3′-diaminobenzidine (DAB) as the chromogen.

### Compartment-specific analysis

Staining intensity was evaluated separately in the following compartments:

- Interstitial compartment.
- Bronchiolar epithelial compartment.

Quantitative analysis was performed using QuPath software (v0.6.0; University of Edinburgh, Edinburgh, UK) and results were expressed as the percentage of positively stained cells.

### Statistical analysis

Data were analyzed using IBM SPSS Statistics for Windows, Version 20.0 (IBM Corp., Armonk, NY, USA) and GraphPad Prism 8 (GraphPad Software, Boston, MA, USA).

Histopathological scores were analyzed using the Kruskal–Wallis test, followed by Bonferroni-adjusted Mann–Whitney U tests for pairwise comparisons.

Immunohistochemical data (percentage of positively stained cells) were analyzed using one-way analysis of variance (ANOVA), followed by Tukey’s post hoc test, after confirming normality with the Shapiro–Wilk test and homogeneity of variance with Levene’s test.

Each dam was considered as an independent experimental unit in the statistical analysis.

A two-sided p value of < 0.05 was considered statistically significant.

## Results

### Histopathological and immunohistochemical findings

Histopathological evaluation showed that the Control, DEX, and BET groups maintained near-normal alveolar architecture with thin septa and no significant inflammatory infiltration. The ERDO group displayed mild interstitial infiltration but retained overall lung structure, suggesting a limited inflammatory response (Fig. 1).

Immunohistochemical analysis revealed distinct expression patterns across groups.

Caspase-3 immunoreactivity was predominantly observed in the bronchiolar epithelium in the DEX and BET groups, whereas minimal staining was detected in the ERDO and control groups. PCNA expression showed nuclear localization, with higher proliferative activity in the DEX and BET groups compared to ERDO and control. ABCA3 expression was most prominent in both interstitial and epithelial compartments in the ERDO group. SFTPA1 demonstrated compartment-specific distribution, with increased interstitial staining in the ERDO group. These quantitative findings are supported by representative immunohistochemical images shown in Figs. 3, 4, 5 and 6.

**Fig. 2:**
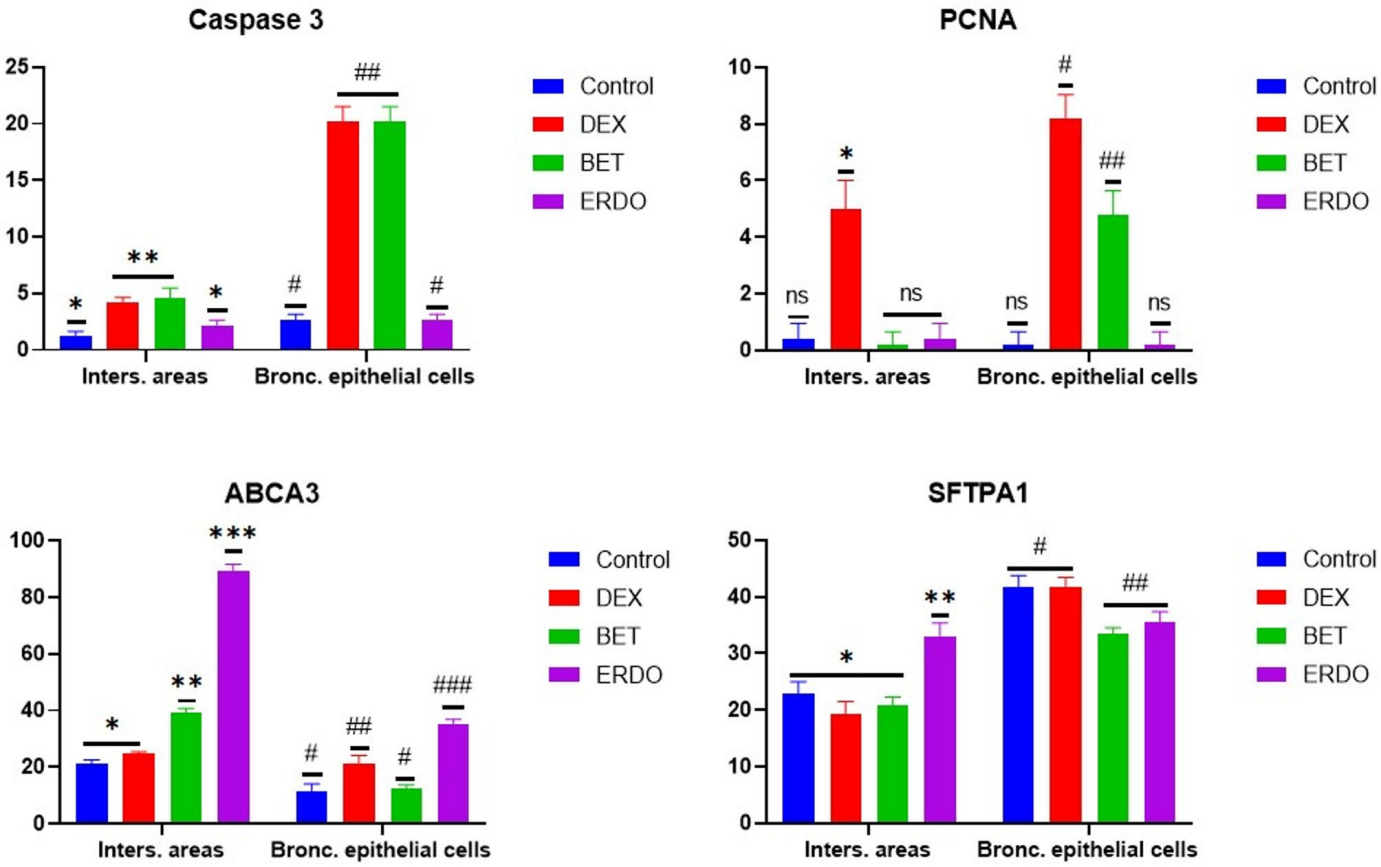
Comparative statistical analysis of immunohistochemical markers (Caspase-3, PCNA, ABCA3, SFTPA1, AQP5, SP-B). Significant intergroup differences are indicated by *, **, ***, #, ##; ns: not significant. Values are expressed as percentage of positively stained cells (%).

Overall statistical comparisons demonstrated significant intergroup differences for Caspase-3, PCNA, ABCA3, and SFTPA1 (*p* < 0.05), while AQP5 and SP-B were negative in all groups (Fig. 2).

**Fig. 3:**
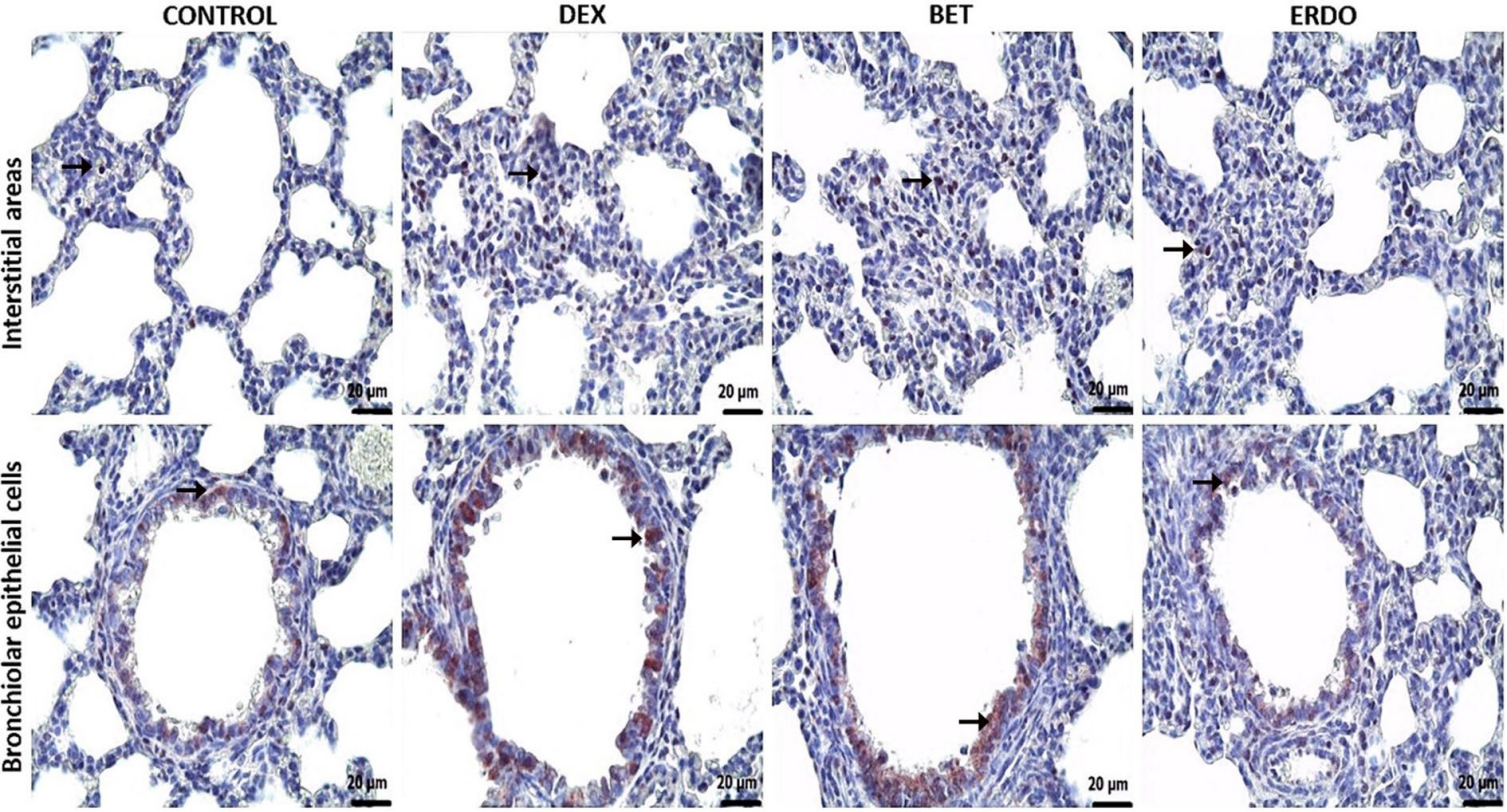
Caspase-3 immunoreactivity across groups. Arrows indicate representative positively stained cells in the corresponding compartments. Bronchiolar sections; IHC. Scale bar: 20 μm.

**Fig. 4:**
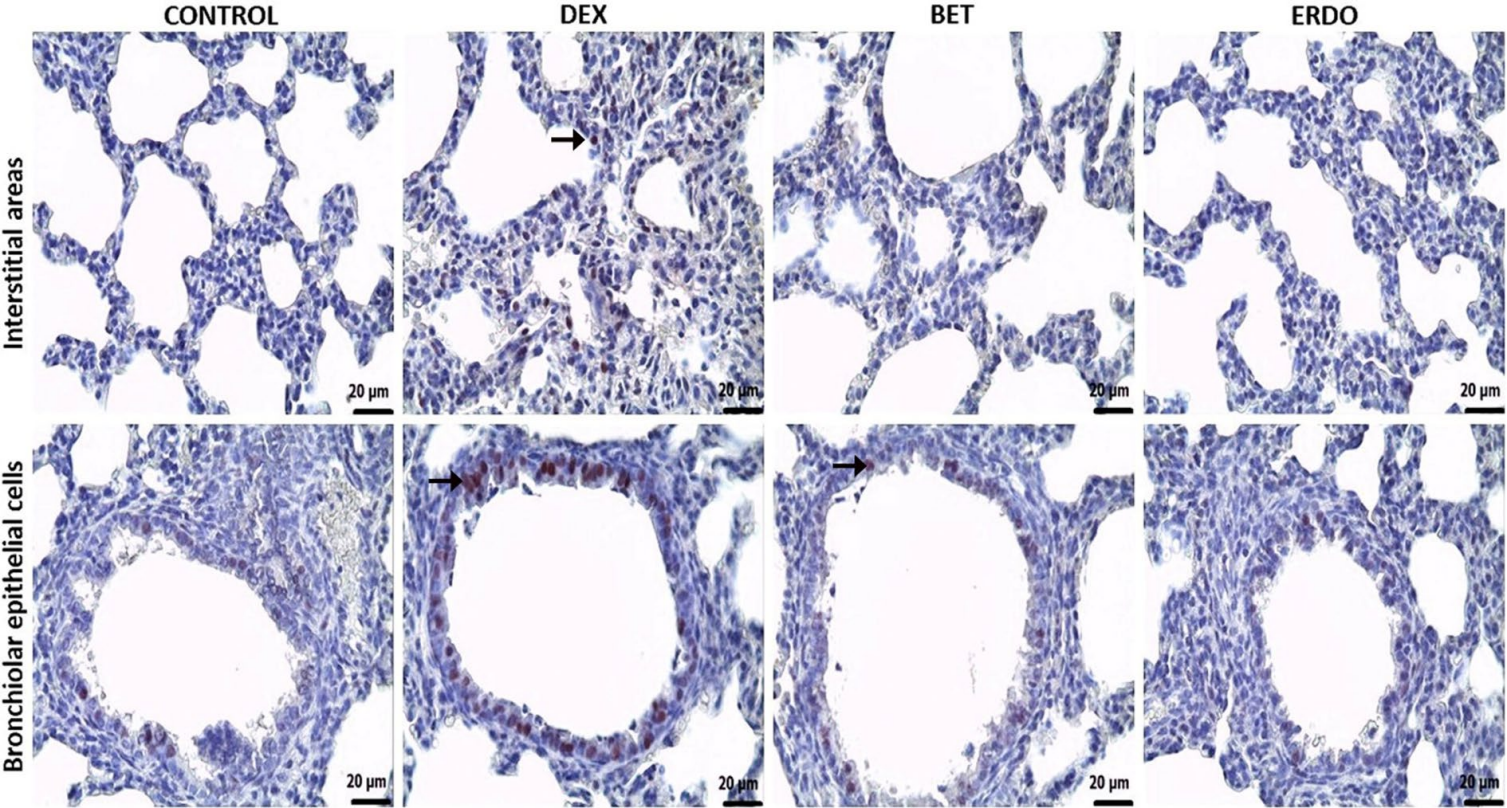
PCNA immunoreactivity showing differential proliferative activity among groups. Arrows denote PCNA-positive nuclei. Scale bar: 20 μm.

Caspase-3 and PCNA were minimally expressed in the Control and ERDO groups but were significantly higher in the DEX and BET groups, indicating increased apoptosis and proliferation due to glucocorticoid exposure (Figs. 3 and 4).

**Fig. 5:**
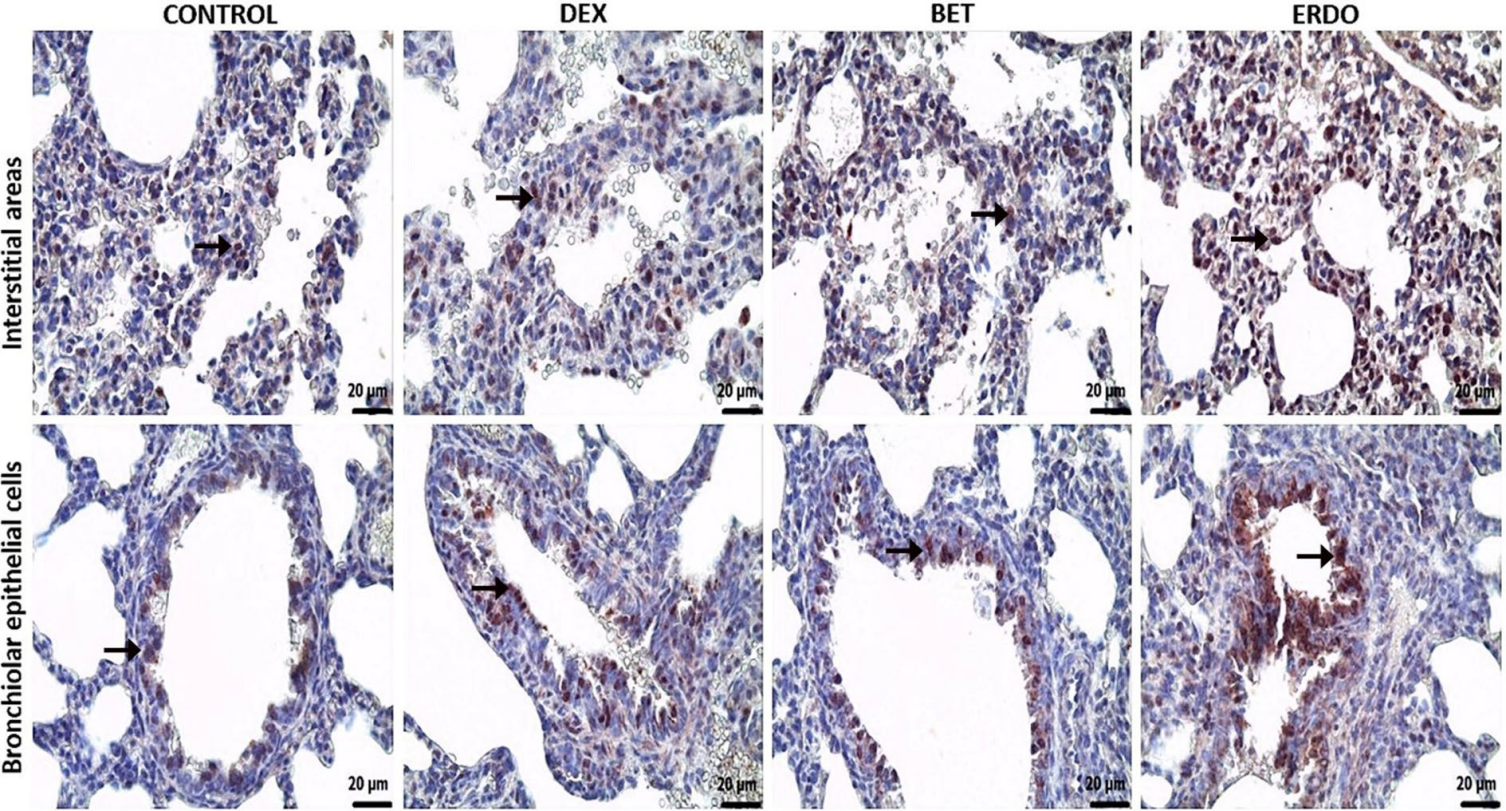
ABCA3 immunostaining demonstrating marked interstitial and epithelial expression in the ERDO group. Arrows indicate representative positively stained cells in the corresponding compartments. Scale bar: 20 μm.

ABCA3 was most strongly expressed in the ERDO group, followed by DEX, with lower levels in BET and Control (Fig. 5).

**Fig. 6:**
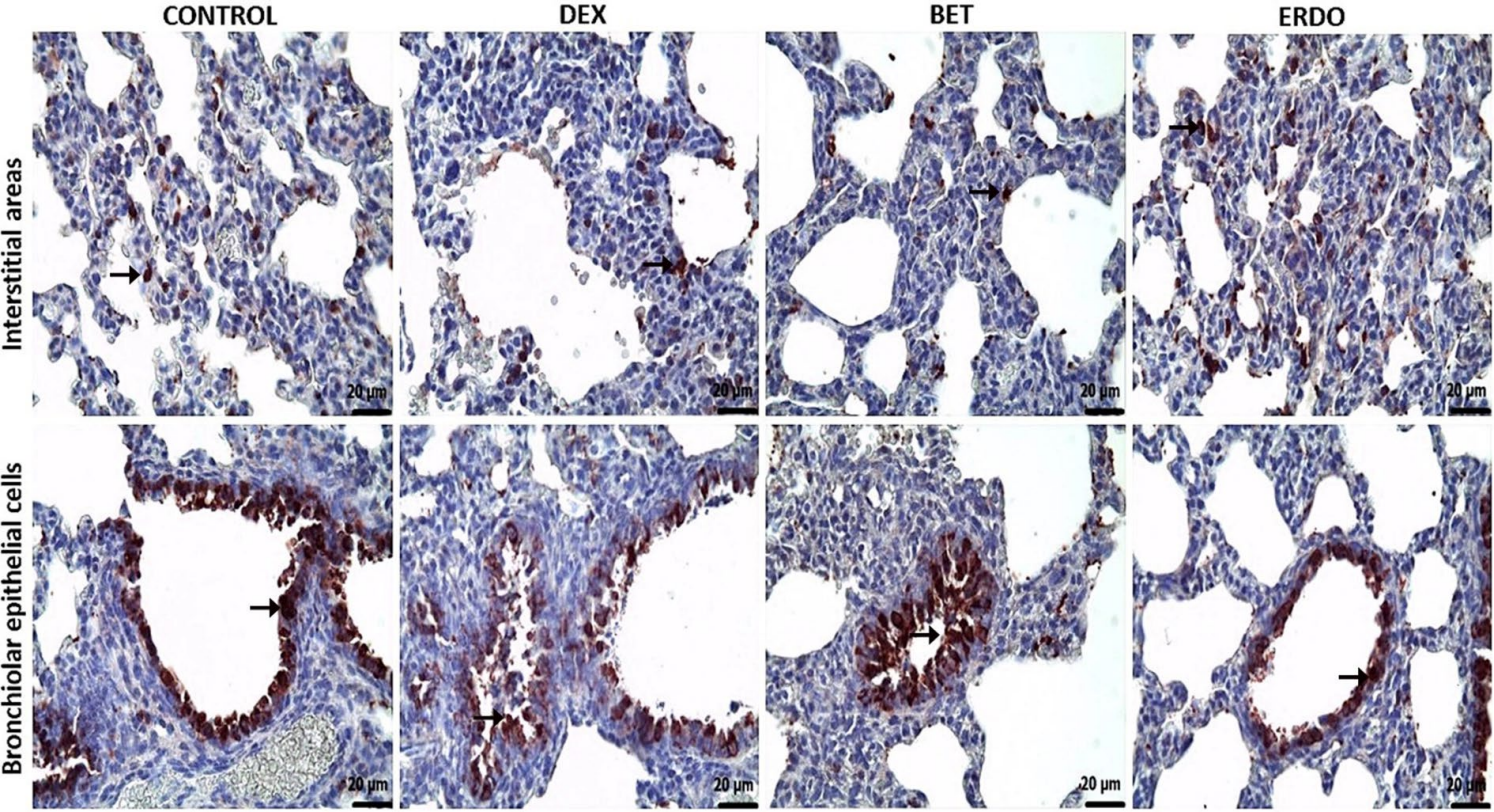
SFTPA1 immunostaining showing group-specific differences in interstitial and epithelial compartments. Arrows indicate representative positively stained cells in the corresponding compartments. Scale bar: 20 μm.

SFTPA1 was more prominent in the interstitial compartment of the ERDO group, while DEX and Control had higher epithelial expression (Fig. 6).

**Fig. 7:**
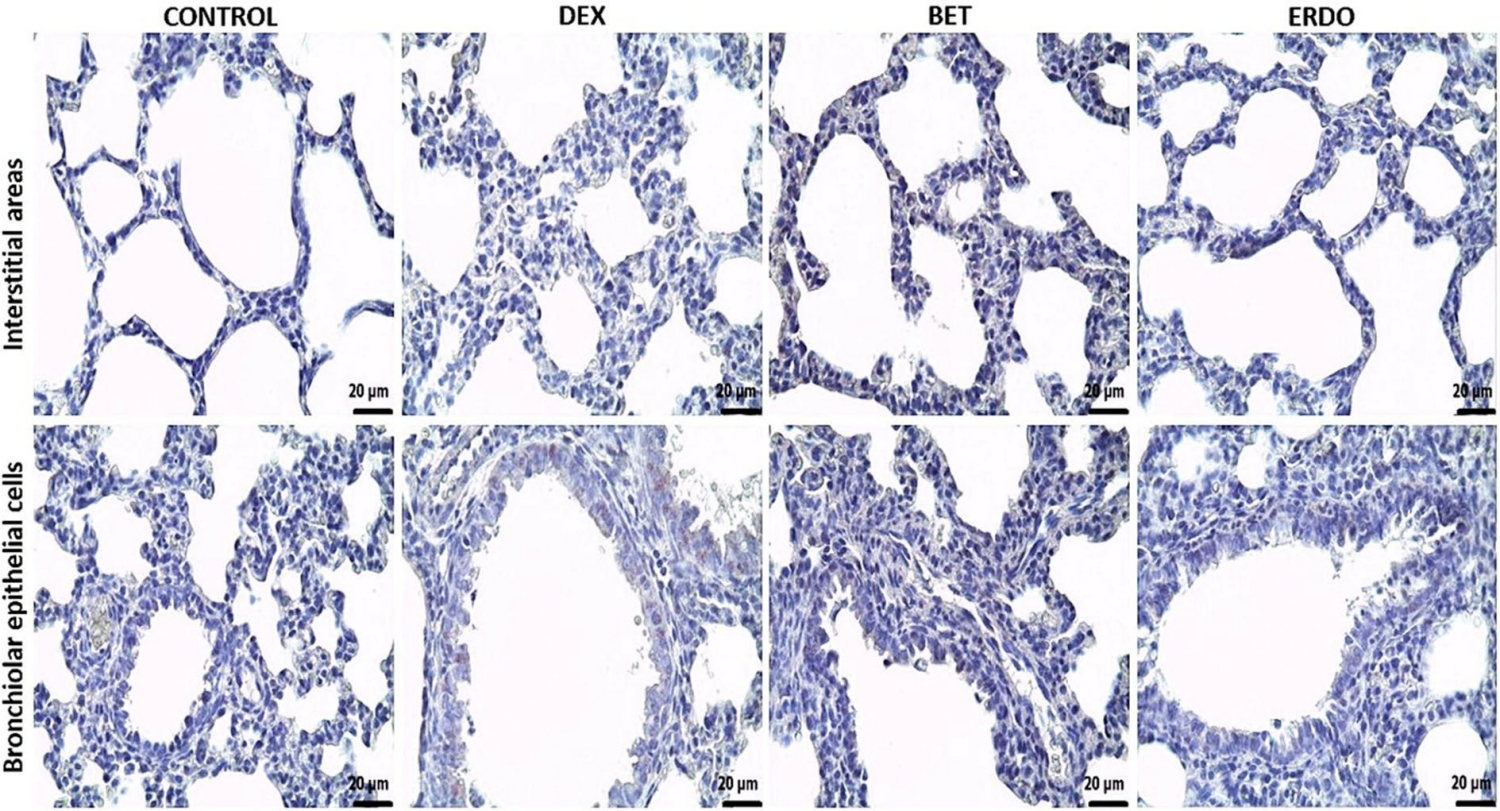
AQP5 immunostaining in bronchiolar and interstitial regions across all experimental groups. No specific AQP5 expression was detected in any group, consistent with the low developmental expression of AQP5 in preterm rat lungs at P5. Arrows indicate representative positively stained cells in the corresponding compartments. Scale bar: 20 μm.

**Fig. 8:**
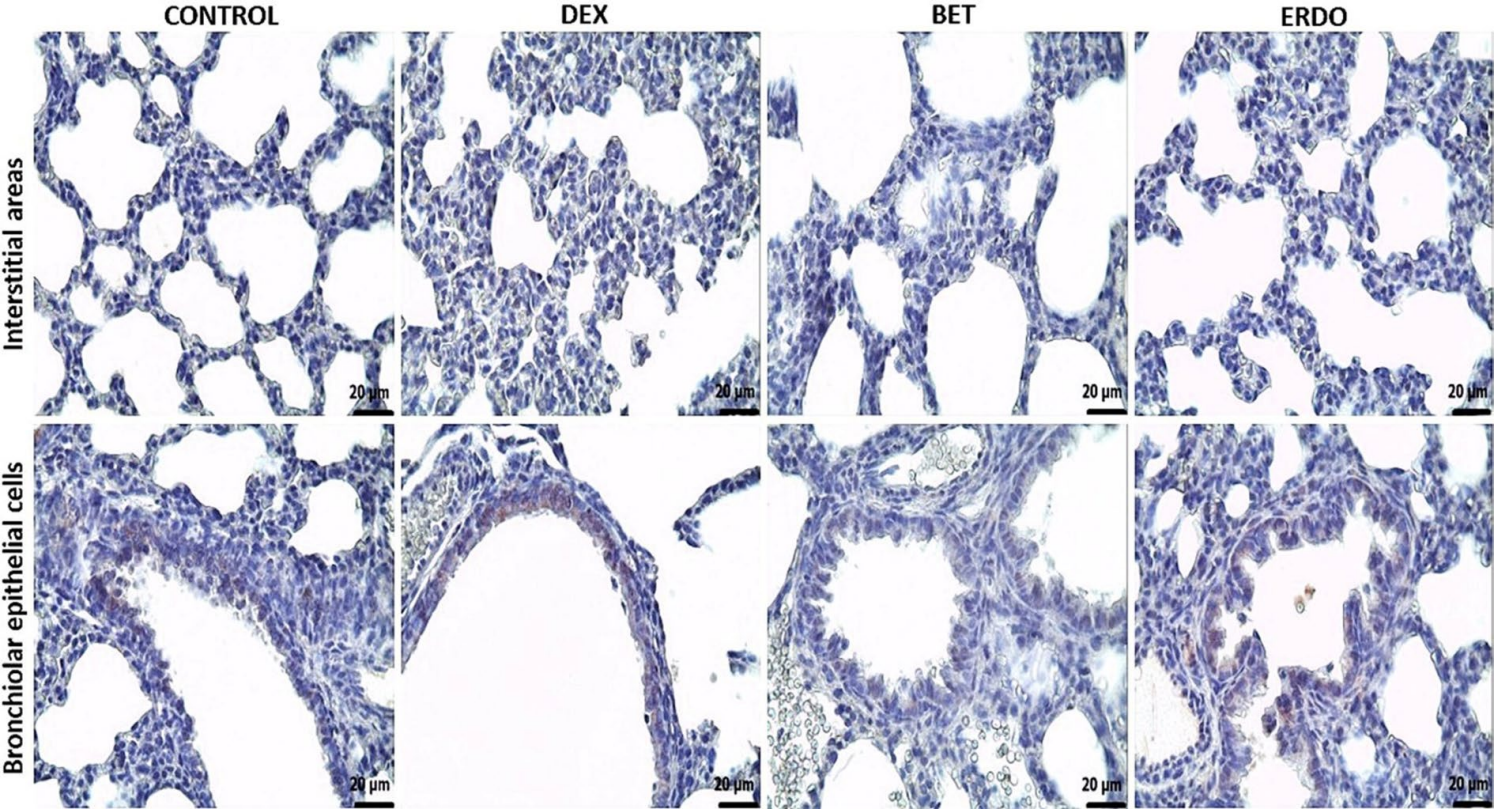
SP-B immunostaining in bronchiolar and interstitial compartments of the neonatal lung. All groups demonstrated complete immunonegativity for SP-B, reflecting the expected immaturity of surfactant protein B expression in early postnatal lungs. Arrows indicate representative positively stained cells in the corresponding compartments. Scale bar 20 μm.

AQP5 and SP-B were not detected in any group, consistent with the expected low levels at this developmental stage (Figs. 7 and 8).

## Discussion

Preterm birth–related respiratory distress syndrome remains a major cause of neonatal morbidity and mortality and is primarily driven by surfactant deficiency and structural immaturity of the lung. Antenatal corticosteroids are still the standard of care to accelerate fetal lung maturation and reduce early respiratory complications. However, concerns about possible long-term cardiometabolic or neurodevelopmental effects, especially with repeated or high-dose exposure, have renewed interest in alternative or adjunctive strategies that might support lung maturation with a more favourable safety profile^19–21^.

More detailed evaluation of immunohistochemical staining patterns revealed marker-specific localization and intensity differences across groups.

In this preterm rat model, dexamethasone and betamethasone produced a nearly normal histological appearance, whereas erdosteine resulted in preserved alveolar structure with mild interstitial mononuclear cell infiltration. These findings indicate that erdosteine does not induce overt structural injury but is associated with a modest inflammatory response. The minimal inflammation observed in the corticosteroid groups aligns with studies showing that antenatal betamethasone reduces inflammatory cell accumulation and stabilises alveolar structure^22,23^. Given that glucocorticoids suppress NF-κB–dependent cytokine release and leukocyte recruitment^24^, the reduced inflammatory scores in DEX and BET are expected. In contrast, the mild infiltration observed in the erdosteine group cannot be further characterised based on the current data and should be interpreted cautiously.

Caspase-3 expression was increased in both the interstitial and bronchiolar epithelium of the corticosteroid-treated groups, consistent with previous ovine and rodent studies reporting steroid-induced epithelial apoptosis and thinning of alveolar septa^23,26^. In contrast, caspase-3 positivity remained low in the erdosteine group, which is in line with reports suggesting that erdosteine and its metabolites may reduce oxidative stress–related apoptotic signalling^14,25,27^. These findings suggest a differential apoptosis profile between treatment groups; however, functional implications cannot be inferred from these data alone.

The PCNA expression pattern further supports this divergence. Dexamethasone showed the highest proliferative activity, followed by betamethasone, whereas erdosteine and control lungs exhibited lower levels. This is compatible with reports that glucocorticoids transiently enhance epithelial proliferation and differentiation before promoting maturation of alveolar structures^28,29^. The relatively modest PCNA positivity in the erdosteine group suggests that its effects may not be mediated through direct stimulation of proliferation.

One of the most notable findings is the ABCA3 expression profile. ABCA3, a key ATP-binding cassette lipid transporter in type II pneumocytes, is essential for phospholipid transport, lamellar body maturation and surfactant homeostasis^30–33^. Dysfunction of ABCA3 is associated with severe neonatal respiratory disease and impaired alveolar stability^32,34,35^. Although corticosteroids have been shown to enhance ABCA3 expression in experimental models^5,36^, our data demonstrated higher ABCA3 staining in the erdosteine group across both evaluated compartments. While this finding may indicate a potential influence on surfactant-related pathways, it should be interpreted cautiously, as immunohistochemical expression does not directly reflect functional surfactant activity or lipid transport.

SFTPA1 displayed a compartment-specific profile. Interstitial staining was most pronounced in the erdosteine group, while epithelial expression was higher in the control and DEX groups and lower in BET and ERDO. SFTPA1 is a major hydrophilic surfactant protein involved in surfactant structure, surface-tension regulation, pathogen opsonisation and immunomodulation^37–39^. Its expression increases during the late canalicular and saccular stages, and experimental models confirm glucocorticoid-driven upregulation of SP-A^40–43^. The distribution observed in this study suggests differences in compartmental expression patterns between treatment groups; however, the functional significance of this finding remains unclear.

AQP5 and SP-B were immunonegative across all groups at postnatal day 5. This is consistent with developmental reports demonstrating that both markers increase closer to term and early postnatally, often remaining low at very early time points, particularly in immature lung models^44–46^. Thus, their absence likely reflects developmental stage rather than intervention-specific suppression.

Taken together, these findings indicate that erdosteine supports preterm lung maturation in a manner partially overlapping with but distinct from classical corticosteroids. Erdosteine increased ABCA3 and interstitial SFTPA1 expression while maintaining low apoptosis and modest proliferation, accompanied by preserved lung architecture. Dexamethasone and betamethasone induced stronger apoptotic and proliferative responses, leading to near-normal histology but relatively lower ABCA3 levels than erdosteine. These findings suggest that thiol-based antioxidant therapy may represent a potential adjunctive approach to surfactant-related pathways, although its clinical relevance requires further investigation.

These findings suggest that thiol-based antioxidant therapy may be associated with modulation of surfactant-related pathways through mechanisms that are biologically distinct from glucocorticoid-driven effects. However, the clinical relevance of these observations remains uncertain and requires further investigation. Future studies should focus on dose optimization, timing of administration, and interaction with standard antenatal corticosteroid regimens in clinically relevant models and, ultimately, in human studies.

This study has several limitations. First, the sample size was relatively small, and analyses were limited to a single early postnatal time point, precluding evaluation of long-term structural and functional outcomes. Second, the study relied on histopathological and immunohistochemical analyses without direct functional assessment of lung mechanics or surfactant activity. Third, inflammatory findings observed in the ERDO group could not be further characterized, as cytokine profiling and immune cell phenotyping were not performed. Fourth, quantitative morphometric analyses such as mean linear intercept (MLI) and radial alveolar count (RAC), which are standard methods for assessing alveolar development, were not included. Finally, additional protein-level validation methods, such as Western blot analysis, were not performed, which may have further strengthened the molecular findings.

Despite these limitations, the present findings provide preliminary experimental evidence that erdosteine modulates selected markers associated with surfactant regulation, including ABCA3 and SFTPA1, and exhibits a distinct apoptosis–proliferation profile compared with dexamethasone and betamethasone. However, these results should be interpreted cautiously and primarily reflect molecular and cellular alterations rather than definitive functional lung maturation. Further studies incorporating functional respiratory assessments, cytokine profiling, morphometric analyses, and long-term follow-up are required to clarify the potential role of erdosteine in perinatal lung development. In addition, evaluation of other surfactant-associated proteins such as SP-C and SP-D, as well as epithelial sodium channel (α-ENaC) expression, may provide further mechanistic insight.

## Conclusions

In this preterm rat model, erdosteine exhibited a maturation profile that was clearly distinct from that of dexamethasone and betamethasone. While classical antenatal corticosteroids generated robust apoptotic and proliferative activity consistent with accelerated structural maturation, erdosteine produced minimal apoptosis, preserved alveolar architecture and a comparatively modest proliferative response. Most notably, erdosteine markedly increased ABCA3 expression and enhanced interstitial SFTPA1 staining, suggesting a potential influence on surfactant-related lipid transport and innate immune pathways. These effects emerged without the degree of tissue remodelling observed in the corticosteroid groups.

Taken together, the data indicate that thiol-based antioxidant therapy may support surfactant biogenesis and early lung maturation through mechanisms partially overlapping with, yet biologically distinct from, glucocorticoid-driven pathways.

This study has several limitations. First, the sample size was relatively small, and analyses were limited to a single early postnatal time point, precluding evaluation of long-term structural and functional outcomes. Second, the study relied on histopathological and immunohistochemical analyses without direct functional assessment of lung mechanics or surfactant activity.

Importantly, molecular validation techniques such as RT-PCR or Western blot analysis were not performed. Therefore, the observed differences in surfactant-associated markers should be interpreted as indicative rather than definitive evidence of altered surfactant synthesis.

In addition, inflammatory findings observed in the ERDO group could not be further characterized due to the absence of cytokine profiling or immune cell phenotyping. Quantitative morphometric analyses such as mean linear intercept and radial alveolar count were also not included.

Finally, the absence of detectable SP-B and AQP5 expression should be interpreted in the context of the developmental stage of the preterm model rather than as a complete lack of surfactant production.

However, these findings are based on histopathological and immunohistochemical analyses and do not provide direct evidence of functional lung maturation. Therefore, the results should be interpreted with caution.

Future studies incorporating molecular validation, dose–response analyses, combined treatment strategies, and long-term functional outcomes will be essential to further define the potential role of erdosteine in preterm lung development.

## Data Availability

The datasets generated and analyzed during the current study are available from the corresponding author on reasonable request.

## Abbreviations

ABCA3: ATP-binding cassette subfamily A member 3
ACS: Antenatal corticosteroids
AQP5: Aquaporin-5
BET: Betamethasone
DEX: Dexamethasone
ERDO: Erdosteine
H&E: Hematoxylin and eosin
IHC: Immunohistochemistry
PCNA: Proliferating cell nuclear antigen
SFTPA1: Surfactant protein A1
SP-A: Surfactant protein A
SP-B: Surfactant protein B
SPSS: Statistical Package for the Social Sciences
